# Improved Localization of Seizure Onset Zones Using Spatiotemporal Constraints and Time-Varying Source Connectivity

**DOI:** 10.3389/fnins.2017.00156

**Published:** 2017-04-06

**Authors:** Juan D. Martinez-Vargas, Gregor Strobbe, Kristl Vonck, Pieter van Mierlo, German Castellanos-Dominguez

**Affiliations:** ^1^Signal Processing and Recognition Group, Universidad Nacional de ColombiaManizales, Colombia; ^2^Medical Image and Signal Processing Group, iMinds Medical IT Department, Ghent UniversityGhent, Belgium; ^3^Laboratory for Clinical and Experimental Neurophysiology, Neurobiology and Neuropsychology, Ghent University HospitalGhent, Belgium

**Keywords:** EEG source connectivity, non stationary brain activity, directed functional connectivity, seizure-onset zone, pre-surgical evaluation

## Abstract

Presurgical evaluation of brain neural activity is commonly carried out in refractory epilepsy patients to delineate as accurately as possible the seizure onset zone (SOZ) before epilepsy surgery. In practice, any subjective interpretation of electroencephalographic (EEG) recordings is hindered mainly because of the highly stochastic behavior of the epileptic activity. We propose a new method for dynamic source connectivity analysis that aims to accurately localize the seizure onset zones by explicitly including temporal, spectral, and spatial information of the brain neural activity extracted from EEG recordings. In particular, we encode the source nonstationarities in three critical stages of processing: Inverse problem solution, estimation of the time courses extracted from the regions of interest, and connectivity assessment. With the aim to correctly encode all temporal dynamics of the seizure-related neural network, a directed functional connectivity measure is employed to quantify the information flow variations over the time window of interest. Obtained results on simulated and real EEG data confirm that the proposed approach improves the accuracy of SOZ localization.

## 1. Introduction

Approximately 70% of epilepsy patients can be adequately treated with antiepileptic drugs. For the remaining 30% that has pharmaco-resistant epilepsy (or refractory epilepsy), other treatment options such as resection surgery or neurostimulation (like deep brain stimulation or vagus nerve stimulation) must be strongly considered. The purpose of resection surgery is to remove the epileptogenic focus, i.e., a limited area of the neural tissue that is sufficient to be eliminated from the brain, leading to seizure freedom in the patient. During a pre-surgical procedure, the effectiveness of epilepsy resection is evaluated by localizing as accurately as possible the epileptogenic focus that overlaps with eloquent cortex grounded in different neuroimaging techniques. Hence, the localization the brain regions where the seizures originate from [termed seizure onset zone (SOZ)] is of utmost importance. To this end, several anatomical and functional imaging techniques have been used, being intracranial EEG the gold standard. Nevertheless, this technique is invasive and implies one surgery more before the SOZ resection should be carried out.

As an alternative in the pre-surgical evaluation of epilepsy, Electroencephalography (EEG) is widely used as a non-invasive neuroimaging technique that measures the brain electrical activity by placing electrodes on the scalp. Accordingly, the source imaging methods are employed, which address the EEG inverse problem, to map the acquired neural data into source space information with the aim to localize the epileptic generators over the patient's cortical surface that is extracted from the available Magnetic Resonance Image (MRI) (Heers et al., 2016).

Although different source imaging methods have been used for SOZ localization (like in Brodbeck et al., 2011; Pellegrino et al., 2016), recent progress in neuroimaging suggests that many distant brain areas must also be involved during seizures. Furthermore, the neural activity related to a single SOZ propagates to other separated brain regions, producing an epileptic network (Pittau et al., 2014). However, the fast non-stationary dynamics inherent to epileptic activity imply abrupt changes of amplitude/frequency of EEG data, making it difficult to discriminate the actual SOZ from the widespread epileptic network activity (Elshoff et al., 2013). One of the promising approaches to solving this problem is the EEG source connectivity analysis that quantifies the activity interactions between distant brain areas (Bastos and Schoffelen, 2015). Regarding the SOZ localization, we explore several scenarios of EEG source connectivity that comprise three main steps: (i) EEG source imaging, (ii) Grouping of the active areas that are more likely to be related to the studied phenomena [termed Regions-of-Interest (ROIs)] and computation of their respective time-courses, and (iii) Assessment of connectivity between ROIs.

In the source estimation step, every one of the active sources belonging to the epileptic network must be spatially localized with enough accuracy to provide a proper SOZ localization (Grosse-wentrup, 2009). Two main restrictions bound this aspect: The common assumption of temporal stationarity that cannot be guaranteed in practice for seizure related neural activity (Giraldo-Suarez et al., 2016), and the appearance of several artifacts during a seizure like muscle contractions or eye blink, which distort even more the source reconstruction and mislead determination of the brain activity foci. Accordingly, the applied source imaging method must take advantage of the outstanding temporal resolution provided by the available EEG data to enhance the epileptic network localization.

In the next step, the quality of the computed ROI set strongly determines the achieved connectivity analysis performance. To this end, we carry out the grouping of active brain sources, which are spatially adjacent and correlated to the studied phenomena, estimating the time-courses that properly describe the temporal patterns emerged in each region. In the space domain, ROIs can be detected, assuming a priori knowledge of their relationship with a given experimental task (e.g., from functional imaging studies or physiological information). Nonetheless, the most widely-used strategy is the ROI detection based on neural activity maps due to prior information is frequently unavailable (Schoffelen and Gross, 2009). In the temporal domain, the time courses are often computed by averaging the dipole time-courses comprised in each ROI (Hassan et al., 2014). Due to the non-stationary behavior of seizure activity, however, the average tends to misrepresent the actual temporal dynamics of the studied region. Hence, the ROI locations and the related time-courses (calculated from the reconstructed sources) must supply enough spatial and temporal resolution to reflect correctly every one of the nodes involved in the epileptic network.

In the last step, the functional connectivity assessment measures the information flow direction between brain regions, i.e., the influence that one neural system exerts over another, either directly or indirectly (Friston et al., 1993). Therefore, the used assessment must properly encode the dynamic changes of the network structure over time (Astolfi et al., 2008; van Mierlo et al., 2013).

This work introduces a new scenario of source connectivity analysis that explicitly extracts temporal, spectral, and spatial information from EEG data to improve the SOZ localization. To this end, we initially carry out the time-varying brain source reconstruction, from which ROIs are localized as the brain areas with strong activity. As a means to compute further the corresponding ROI time courses, we also propose a new approach that relies on accurately tracking the non-stationary temporal dynamics in spatially focal brain areas. Aiming to encode the temporal dynamics of the underlying neural networks accurately, a directed functional connectivity measure is employed to quantify the information flow variations over the time window of interest. Obtained results on simulated and real EEG confirm that the proposed approach allows localizing SOZ targets accurately.

The rest of the manuscript is organized as follows: In Section 2, we describe the proposed source connectivity method. So, we outline the proposed EEG inverse problem solution based on temporal constraints, from which the ROI set and its corresponding time courses are computed so that they track the non-stationary dynamics of the epileptic events. Also, we describe the introduced directed functional connectivity measure. Further, Section 3 describes the experimental setup designed for comparing the proposed methodology with some state-of-the-art approaches, varying the channel number of the EEG systems. We also apply our method to real-world ictal EEG recordings in three patients. In Section 4, we address the interpretation of obtained results, remarking our contribution in Section 5.

## 2. Methods

### 2.1. Estimation of cortical sources

Characterization of connectivity patterns between pairs of ROIs necessarily requires that electrophysiological signals be assessed in the source space (Brookes et al., 2014). That is, by solving the EEG inverse problem, the scalp neural field data should be projected into the source space through a known gain matrix, commonly known as the lead field matrix. Mathematically, this formulation can be represented through the linear model ***Y***=***MJ***+Ξ, where ***Y***∈ℝ^*C*×*T*^ is the EEG data of *C* sensors and *T* time samples, ***J***∈ℝ^*D*×*T*^ is the amplitude of *D* current dipoles distributed through the cortical surface with fixed orientation perpendicular to it. Moreover, ***M***∈ℝ^*C*×*D*^ is a gain matrix (also known as the lead field matrix) that relates both sensors and the sources. Sensor noise and uncertainty on the propagation model are represented by **Ξ**∈ℝ^*C*×*T*^. Here, the source space projection is performed via the Multiple Sparse Prior (*MSP*) algorithm that includes a two level-hierarchical parametric empirical Bayesian model, for which the source covariance is the weighted sum of multiple empirical priors with compact spatial support so that they are independent but locally determined on the basis of brain anatomy (Strobbe et al., 2014). Hence, the weighted parameters are optimized just based on the available data by maximizing the so-termed Free Energy cost function and using standard variational schemes such as Expectation Maximization (Wipf et al., 2010).

### 2.2. Detection of regions of interest

Since the quality of derived time-courses is a critical issue to represent the information flow throughout the brain volume, the EEG source connectivity analysis demands an accurate identification of the brain areas involved in the investigated neurological problem. Hence, we focus on building a matrix ***L***∈[0 1]^*D*×*R*^ that holds the membership, indicating whether a dipole belongs (i.e., the value is 1) or not (0) to each one of *R* detected ROIs. Built on the computed neural activity maps (inverse solution) and taking into account the neighboring dipole information (derived from the forward solution), we define the ROI set by clustering all active brain sources that are spatially adjacent.

As a useful index of the brain activity captured within a given time-window, we rely on the following estimate for the neural activity power:

(1) $$\xi =(J\circ J)1_{T},\;\xi \in {\mathbb{R}}^{D\times 1}$$

where notation ∘ stands for the Hadamard product and **1**_*T*_ is the all-ones vector sizing (*T*×1). Besides, the adjacent brain areas are determined through the introduced membership matrix Λ∈[0 1]^*D*×*D*^ that limits each dipole neighborhood. Thus, a matrix element Λ(*d, d*′) is assigned the value 1 if a couple of dipoles *d* and *d*′ are nearby. Otherwise, it is zero.

From the above, we develop the following procedure for ROI detection: Initially, we scan the activity reconstruction over the brain cortex to detect the most powerful active dipole, denoting as d^*^ its cardinal number in the vector ξ. So, the first ROI is the d^*^ column of Λ since it holds the position of neighboring dipoles, becoming the first column of ***L***. Then, the activity reconstruction scan is performed again, excluding the active dipoles that belong to the first ROI, to generate the second ROI (second column of ***L***). Likewise, this procedure is repeated over and over again until all active dipoles are assigned to the ROI set, yielding automatically the amount of extracted ROIs *R*.

To encode the activity inhomogeneity on space within each ROI, every column of ***L*** is pointwise multiplied by the power vector **ξ**, resulting in the spatial basis matrix $\Phi_{R}\in {\mathbb{R}}^{D\;\times R}$, so that their columns **ϕ***r*∈ℝ^*D*×1^, ∀*r*∈*R*, hold small clusters (or patches) that are compactly distributed over space. This procedure is carried out as below:

(2) $$\phi_{r}=l_{r}\circ \xi ,\forall r\in R,$$

### 2.3. Estimation of ROI time-courses

The performance of the connectivity analysis is highly influenced by the time-courses obtained from the ROI set. Then, the temporal non-stationarity (inherent to the brain activity) must be carefully considered, as it is critical for a correct estimation of these time-courses. Therefore, we propose to model the current density as a linear combination of the neural activity within each ROI, as follows:

(3) $$J=\Phi_{R}H,$$

where ***H***∈ℝ^*R*×*T*^ holds the coefficients to be estimated that reproduce the temporal dynamics of each single ROI, so that *r*-th row of ***H*** includes the temporal patterns (time-courses) of the respective *r*-th ROI. In this regard, estimation of ***H*** is carried out by minimizing the following cost function:

(4) $$\hat{H}=\underset{H}{\mathrm{argmin}}\left\{||Y-M\Phi_{R}H|{|}_{F}^{2}+\Theta \left(H,\gamma \right)\right\},$$

where the added functional term Θ(***H***, **γ**)∈ℝ^+^ holds the constraints imposed upon the vector of regularization parameters **γ**.

In order to improve the estimation of connectivity networks, we enhance the temporal resolution, encouraging the ROI time-courses to be sparse and temporally homogeneous. To this end, we include the fused Lasso (FL) regularization penalty as follows (Tibshirani et al., 2005):

(5) $$\Theta (H;\gamma_{s},\gamma_{t})=\gamma_{s}\sum_{\tau =1}^{T}||h_{\tau }|{|}_{1}+\gamma_{t}\sum_{\tau =2}^{T}||h_{\tau +1}-h_{\tau }|{|}_{1},$$

where ***h***_τ_∈ℝ^*R*×1^ is the τ-th column of ***H***, $\gamma_{s}\in {\mathbb{R}}^{+}$ and $\gamma_{t}\in {\mathbb{R}}^{+}$ are the regularization parameters over the space and time domains, respectively. Notation || · ||_*p*_ stands for the *L*_*p*_-norm. Hence, we expect the first penalty term in Equation (5) to foster the solution sparseness by assigning a high penalty cost to the matrices with large absolute values, and, thus, effectively shrinking the matrix elements toward zero. Accordingly, we identify in τ-th column of ***H*** a subset holding the most active ROIs at the τ-th time sample. In turn, the second penalty term in Equation (5) promotes the temporal homogeneity by penalizing the difference between consecutive time samples, assuming the measured time-courses set to behave constantly within each temporal neighborhood. Nonetheless, the penalty function enables also encoding the temporal variations of neural activity contained over larger time horizons. Note that the optimization of Equations (4) and (5) is carried out via FISTA algorithm as detailed in Chen et al. (2012).

### 2.4. Assessment of pairwise connectivity between ROIs

We employ the directed functional connectivity analysis to refine the actual contribution of the estimated ROI set, i.e., the driver behind the epileptic network. Though several strategies have been developed to investigate the influence that a neural systems exerts over another, we use a multivariate measure to estimate the connectivity between ROIs, based on the Granger causality. Thus, under the assumption that temporal dynamics of ROI time-courses mostly determine the time-varying outcomes of the connectivity analysis, the spectrum-weighted adaptive directed transfer function (*swADTF*) is performed because of its ability to deal with non-stationary signals (van Mierlo et al., 2013). To this end, we initially fit a time-varying multivariate autoregressive (TVAR) model in the estimated ROI time-courses set as follows:

(6) $$c_{t}=\sum_{p\;=\;1}^{P}A_{t}^{p}c_{t\;-\;p}+e_{t},$$

where *P* is the model order, $A_{t}^{p}\in {\mathbb{R}}^{R\times R}$ is the coefficient matrix for delay *p* at time instant *t*, and $e_{t}\in {\mathbb{R}}^{R\times 1}$ the uncorrelated white noise. The model coefficients are estimated using the Kalman filter algorithm, so that they describe the directional information flow between the different signals and may change over time, making the model time-variant.

Then, the *swADTF* at time *t* (denoted as ζ(*t*)∈ℝ^+^) is calculated as follows:

(7) $$\zeta_{ij}(t)=\frac{\sum_{f\in \Delta f}|\delta_{ij}\left(f,t\right){|}^{2}\sum_{r\in R}|\delta_{jr}\left(f,t\right){|}^{2}}{\sum_{k\in R}\sum_{f^{\prime }\in \Delta f^{\prime }}|\delta_{ik}\left(f^{\prime },t\right){|}^{2}\sum_{r^{\prime }\in R}|\delta_{kr^{\prime }}\left(f^{\prime },t\right){|}^{2}},$$

where $\delta_{ij}\left(f,t\right)\in {\mathbb{R}}^{+}$ is the *i, j*-th time-varying coefficient that describes the information flow from the *j*-th ROI to *i*-th ROI at frequency *f* and time *t*, the coefficient set {δ_*ij*_(*f, t*):∀*i, j* = 1, …, *R*} forms the transfer model matrix $\Delta \left(f,t\right)={\left(F\{A_{t}^{p}\}\right)}^{-1}$, and Δ*f*∈ℝ^+^ is a predefined frequency band of neural activity. Notation F{·} stands for the Fourier Transform. Because of the *swADTF* normalization, the sum of information flow into the ROI *i* is equal to one at each time moment, i.e., $\underset{j\in R}{\sum }\zeta_{ij}\left(t\right)=1$. With the aim of choosing the epileptic network driver, we develop the following procedure: We initially sum the *swADTF* values computed for the connectivity between each *j*-th ROI and the remaining ROI set over time (termed outdegree $\eta_{j}\in {\mathbb{R}}^{+}$) as follows:

(8) $$\eta_{j}=\sum_{r\in R,\;t\in T}\zeta_{rj}(t).$$

Then, the ROI with the highest η_*j*_:∀*j* is determined as the seizure onset zone since the outdegree measures the total outgoing information flow.

## 3. Experiments

**Figure 1:**
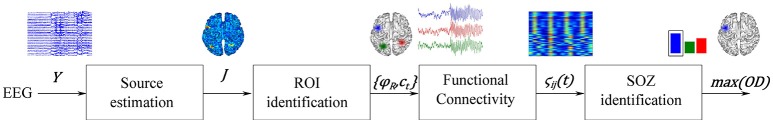
**Illustration of the proposed source connectivity analysis steps to identify the SOZ in epilepsy recordings**. Second box comprises steps (ii) ROI identification and (iii) ROI time courses estimation.

The proposed methodology for localization of seizure-onset zones of brain neural activity, which is based on spatiotemporal constraints and time-varying source connectivity analysis, involves the following four stages (see Figure 1): (i) Estimation of cortical sources from the scalp EEG measurements, relying on each investigated inverse solution method; (ii) Identification of regions of interest, for which several strategies are considered; (iii) Estimation of ROI time-courses, and (iv) Assessment of the pairwise connectivity between selected ROIs to perform SOZ detection. To show some performance examples of the tested methods, the SOZ localization is assessed on two neural activity datasets: one simulated and another obtained from a real-world application.

### 3.1. Performance examples on simulated EEG dataset

In practice, the quality assessment of inverse solutions and connectivity analysis is carried out using simulated sets of EEG recordings, for which the underlying brain activity is known. In this regard, we construct a set of simulated data to test whether the proposed methodology enables properly to localize the seizure onset zone of an epileptic network. To this end, we generate an epileptic network comprising three nodes, following the minimal realistic case of brain interaction to assess ESI and connectivity algorithms subjectively, holding the following guidelines (Haufe and Ewald, 2016): (i) A realistic forward model, (ii) The presence of interacting sources exerting time-delayed influence on another, (iii) Interactions, being confined to a narrow frequency band, (iv) Realistic source locations, being confined to the cortical manifold, (v) Variable locations, different spatial extents, and depths of the sources, (vi) The presence of independent background brain processes with 1/*f* (pink noise) spectra, and (vii) The presence of measurement noise at different SNR levels.

**Figure 2:**
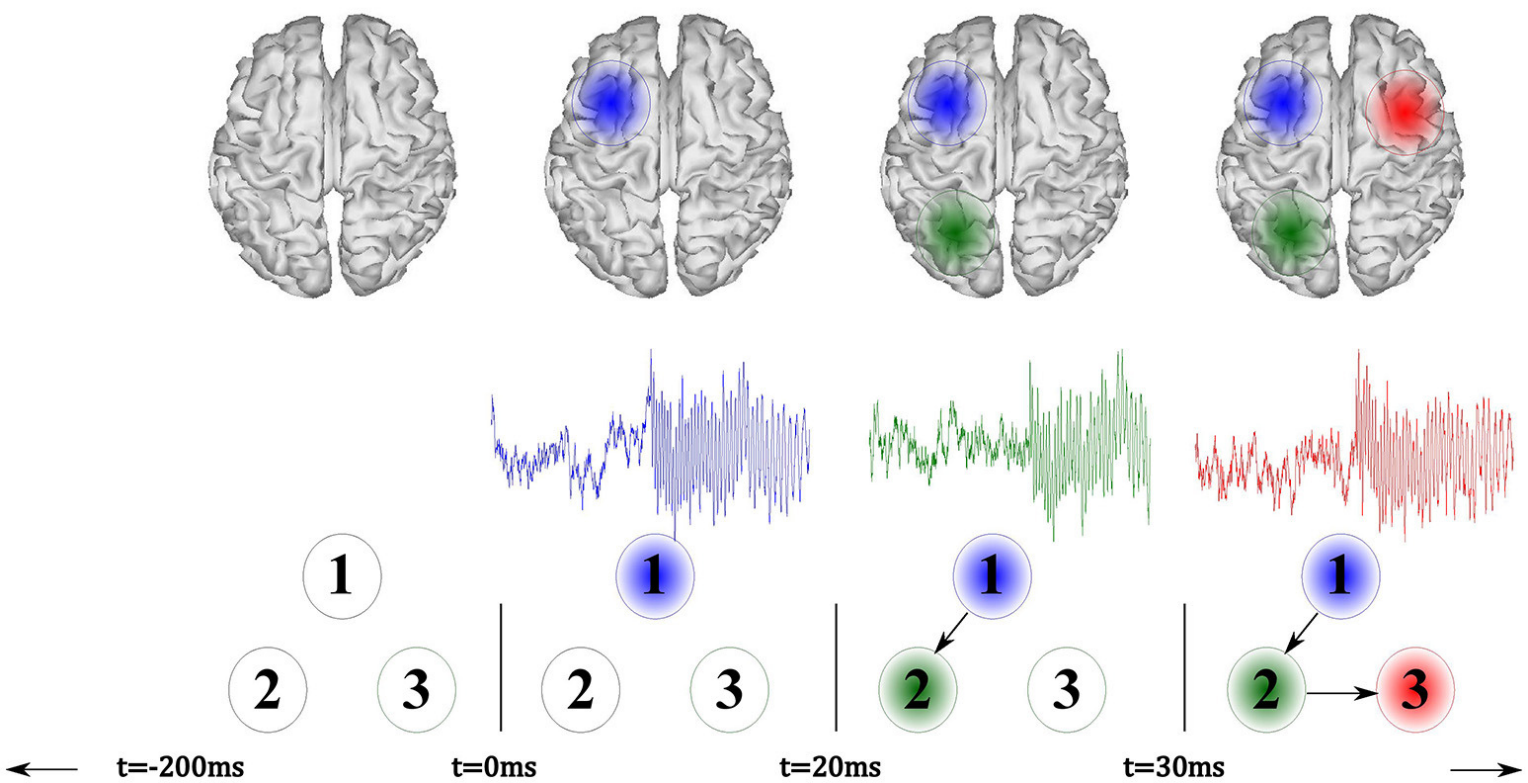
**Illustration of the simulated model that incorporates three signals, where a seizure onset is simulated at ***t*** = 0 ms (blue)**. Afterward, the model reproduces a fast signal propagation.

As shown in Figure 2, the seizure activity is modeled by a sinus function, sampled at 256 Hz, having frequency content that smoothly varies over time from 12,Hz at *t* = 0 ms to 8 Hz within the interval lasting *t* = 3, 000 ms. The simulated seizure placed in the seizure onset zone starts at *t* = 0 ms (the time-series marked in blue). Then, at time *t* = 20 ms, the seizure propagates to the signal 2 (green line) with a time delay of 0.02 s, resembling an interaction of the sources exerting a time-delayed influence. At the end, at *t* = 30 ms, the seizure moves to signal 3 (red line) with a delay of eight samples.

The simulated source activity is mapped to the EEG sensor space using a realistic volume conductor model. The required human head model is constructed from a tessellated surface of the gray-white matter interface with 8, 196 vertices (that is, the possible source localizations), with source orientation fixed orthogonal to the surface. Also, the lead fields are calculated using the Boundary Element Method that simulates a volume conductor with a mean distance between neighboring vertices adjusted to 5 *mm*. Then, each EEG is calculated by multiplying the simulated brain activity to the lead field matrix. As a result, we generate a 128-channel synthetic EEG, for which the electrodes are arranged by the standard 128 BIOSEMI system.

During simulation, we also analyze the performance of the proposed SOZ localization approach when using a lower number of EEG channels. To this end, the 32 closest channels to the 32-channels BIOSEMI system are chosen from the simulated EEG recordings above-described.

For the sake of modeling real-world scenarios of acquired data, the Signal-to-Noise Ratio (SNR) between the epileptic activity and background noise is adjusted to 5 dB. Also, we set the background noise added to the signals to be contained in the 1/*f* spectral behavior. Moreover, the measurement noise (i.e., sensor level) is added to the simulated EEG dataset, providing the following SNR levels: −5, 0, 5, 10 and 15 *dB*. At each noise level, we perform 100 simulation runs having different random source locations.

As pictured in Figure 2, the active sources (marked as blue, green, and red patches) are so placed in the surface cortex locations as to enhance the visual interpretation of the experiment. Nevertheless, the sources may appear in deeper locations of the brain cortex and with lower distances among them during each simulation run.

#### Benchmarking scenarios for SOZ localization

In the case of the simulated EEG dataset, two methods of inverse solution are considered: the baseline *MSP* approach that assumes the stationarity of the tested EEG recording set, and a temporally adaptive-MSP (noted as *taMSP*) that allows extracting the non-stationary dynamics through a stationary-wise analysis. Thus, *taMSP* consists in solving the inverse problem, breaking the EEG analysis time window into several non-overlapped sub-windows so that a piecewise stationary estimation of the brain activity is performed. The length of the sub-windows ***W*** is chosen to span the entire analyzed frequency rank under the rule ***W***>*F*_*r*_/*Fs*, being *F*_*r*_ the smallest considered frequency, and *F*_*s*_ the sample rate. To cover the whole frequency rank and to compute few inverse solutions, we fix the sub-window length to 1 s.

The MSP algorithm has been implemented using the academic software SPM (freely available at^1^) under the standard parameters, including a spatial dictionary comprising 512 basis as to cover the whole cortical surface.

As regards to the neighborhood-based ROI identification, we produce an ROI set spatially compacted, according to the neighboring information encoded in Λ. Furthermore, a threshold is fixed to remove peaks with power values below 5% of the maximum peak, intending to avoid noisy local maxima biasing the results (i.e., only the top 95% of peaks are rated). Thus, we explore two alternatives to estimate the time-courses: the computation of time-varying parameters that belong to each ROI in conformity with Section 2.4, and the estimation by averaging the time-courses within each ROI.

Further, we explore two contrasting strategies for SOZ localization: (i) Power-based detection that picks the ROI with the highest power; and (ii) Connectivity-based detection that picks the ROI with highest association activity (i.e., outdegree) over the time, relying on the *swADTF* measure as a time-varying connectivity measure. To this, the TVAR coefficients (order 10), modeling the ROI time-courses within the frequency band from 0 to 30 Hz are estimated using the Kalman filter algorithm, for which the update coefficient is set to 10^−3^ as suggested in van Mierlo et al. (2011).

Summarizing, we work out four scenarios for considering the influence of each processing stage as is shown in Table 1, displaying the benchmarking scenarios according to their ability to manage the contemplated non-stationary source activity. The proposed methodology (termed *taMSP-FL-Conn*) incorporates the brain activity dynamics in each stage, namely: time adaptive source estimation, calculation of ROI time courses with spatiotemporal constraints, and a time-varying connectivity measure to implement the SOZ localization. As a simplified alternative, the scenario *taMSP-Pow* performs the SOZ detection based on the power criterion. The other tested scenarios are *MSP-Av-Conn* includes time varying information solely in the connectivity measure, and *MSP-Pow* that does not include any information about non-stationary at all. Consequently, the tested scenarios allow comparing a couple of situations: (i) whether the stochastic assumption (stationary or no stationary EEG recordings) helps to increase the capability of the methods to estimate the driver network location (in this case, SOZ); and (ii) whether the connectivity analysis enables providing extra information compared with the source estimation procedure alone.

**Table 1:** **Benchmarked scenarios of source connectivity analysis tested for SOZ localization**.

**Notation**	**Inverse solution**	**Time-course estimation**	**Onset detection**
*taMSP-FL-Conn*	*taMSP*	*Fused Lasso*	*swADTF*
*taMSP-Pow*	*taMSP*	–	*Power*
*MSP-Av-Conn*	*MSP*	*Average*	*swADTF*
*MSP-Pow*	*MSP*	–	*Power*

*For all the scenarios, the ROI identification is carried out by the neighborhood-based method described above*.

#### Results of performed SOZ localization

We contrast the above-mentioned benchmarked scenarios of source connectivity analysis using the misalignment of SOZ localization as the performance measure that computes the minimum Euclidean distance from all dipoles, which belong to the ROI labeled as one SOZ and measured to its actual simulated position. Consequently, the lower the misalignment, the better the SOZ localization. Even that there are more reliable measures (like the earth-movers distance), the baseline Euclidean distance is widely used to assess the ESI performance and connectivity methods of SOZ detection as reported in Strobbe et al. (2016). Unlike the Geodesic-based metrics, this uncomplicated distance can be easily extrapolated for applying on real data as it does not demand a ground truth source distribution, being more relevant to infer at the intra-subject level in three-dimensional anatomical spaces (Henson et al., 2007).

**Figure 3:**
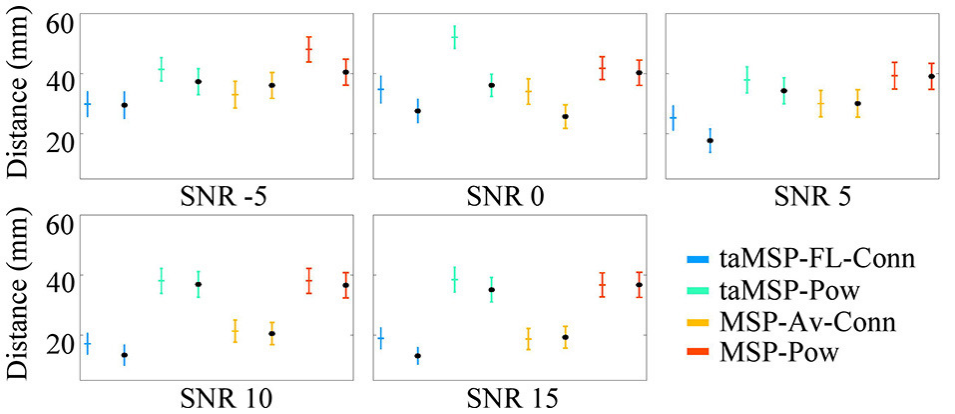
**Precision of the tested methodologies of SOZ location under several levels of noise**. The corresponding color marker for each scenario (i.e., blue for taMSP-FL-Conn) indicates the model with 32 channels, and the black marker (^*^) indicates the model with 128 channels.

Figure 3 displays the mean and standard deviation of the misalignment, which is estimated after 100 trials at each SNR value in either EEG system configuration, namely, 32 and 128 channels (the last configuration is marked with a black dot). As expected, the *MSP-Pow* method that estimates the SOZ without including any temporal information yields the highest misalignment rate (close to 40 mm from the actual SOZ location) within all tested SNR values. Likewise, the SOZ localization does not improve notably if just the temporal information is involved in the cortical source estimation stage (like in *taMSP-Pow*), obtaining misalignment rates very similar to the case of *MSP-Pow*. By contrast, the incorporation of temporal information in the connectivity measure (like in *MSP-Av-Conn*) improves the SOZ localization accuracy in about five points in comparison to *MSP-Pow* and *taMSP-Pow* that do not apply any temporal knowledge. Furthermore, the SOZ localization becomes outstanding when temporal information is included at each stage of the source connectivity method, as in *taMSP-FL-Conn*, reaching misalignment average rates close to 25 mm at low SNR values, and about 10 mm for high SNR values.

Nevertheless, as weakness, the proposed *taMSP-FL-Conn* method seems to be the most sensitive to the electrode number, with differences of about 5 mm between both EEG setups (32 and 128). Apparently, the lower the number of electrodes, the worst the characterization of the temporal dynamics, producing misleading ROI locations distant from the actual SOZ location. Even so, the achieved results by *taMSP-FL-Conn* and 32 channels overcome most of the results accomplished by the remaining approaches having 128 channels.

Aiming to contrast the proposed methodology against the considered training scenarios, we quantify the statistical difference regarding the precision to show whether the taMSP-FL-Conn method achieves misalignment values that are lower than the ones obtained by the remaining approaches. To this end, we use a two-sample *t*-test, for which the null hypothesis states that there are not significant differences between the referent *taMSP-FL-Conn* and each compared scenario in terms of the misalignment mean values. Otherwise, the alternative hypothesis states that our mean misalignment value is lower than the average population estimates performed by the other source connectivity scenarios. This analysis is individually carried out for each setup of acquisition EEG system, that is, 32 and 128-channels data.

As shown in Table 2 for the case of 128-channels setup, *taMSP-FL-Conn* has significant differences in performance with *taMSP-Pow* and *MSP-Pow*, achieving *p*-values lower than 0.01, for all the tested SNR levels. However, there are not significative differences with *MSP-Av-Conn*. In the case of 32-channels setup, however, the *taMSP-FL-Conn* scenario achieves significant differences with all the comparing approaches for 5, 10, and 15 *dB*. Therefore, although *taMSP-FL-Conn* is the most sensitive method for the EEG configuration (number of electrodes), results in Figure 3 and Table 2 show that it allows localizing the SOZ target most accurately than other considered source connectivity scenarios.

**Table 2:** **Performed ***t***-test for computing the significant differences between the referent ***taMSP-FL-Conn*** and each compared scenario of connectivity analysis**.

***SNR***	**Scenario**	**128 Electrodes**			**32 Electrodes**		
		***h***	***t***	***p***	***h***	***t***	***p***
−5	*taMSP-Pow*	1	−2.5609	0.0056^**^	0	−1.5938	0.0563
	*MSP-Av-Conn*	0	−0.6601	0.255	0	−1.3455	0.0900
	*MSP–Pow*	1	−3.8722	0.0001^**^	1	−2.2304	0.0134^*^
0	*taMSP-Pow*	1	−3.8027	0.0001^**^	1	−1.9947	0.0237^*^
	*MSP-Av-Conn*	0	0.1459	0.5579	0	0.4192	0.6622^**^
	*MSP-Pow*	0	−1.5223	0.0648	1	−2.7921	0.0029^**^
5	*taMSP-Pow*	1	−2.6714	0.0041^**^	1	−3.591	0.0002^**^
	*MSP-Av-Conn*	0	−0.9986	0.1596	1	−2.591	0.0052^**^
	*MSP-Pow*	1	−2.9329	0.0019^**^	1	−4.6339	0^**^
10	*taMSP-Pow*	1	−4.5861	0^**^	1	−5.6223	0^**^
	*MSP-Av-Conn*	0	0.0642	0.5256	1	−1.7126	0.0442^*^
	*MSP-Pow*	1	−4.2407	0^**^	1	−5.9189	0^**^
15	*taMSP-Pow*	1	−4.8476	0^**^	1	−5.4617	0^**^
	*MSP-Av-Conn*	0	−1.041	0.1496	1	−1.8083	0.0300^*^
	*MSP-Pow*	1	−4.8289	0^**^	1	−5.4348	0^**^

*, ** *indicates p-values lower than 0.05 and 0.01, respectively. h denotes the decision for the null hypothesis, h is 1 if the test rejects the null hypothesis at the 5% significance level and 0 otherwise, and t is the t-statistic value*.

**Figure 4:**
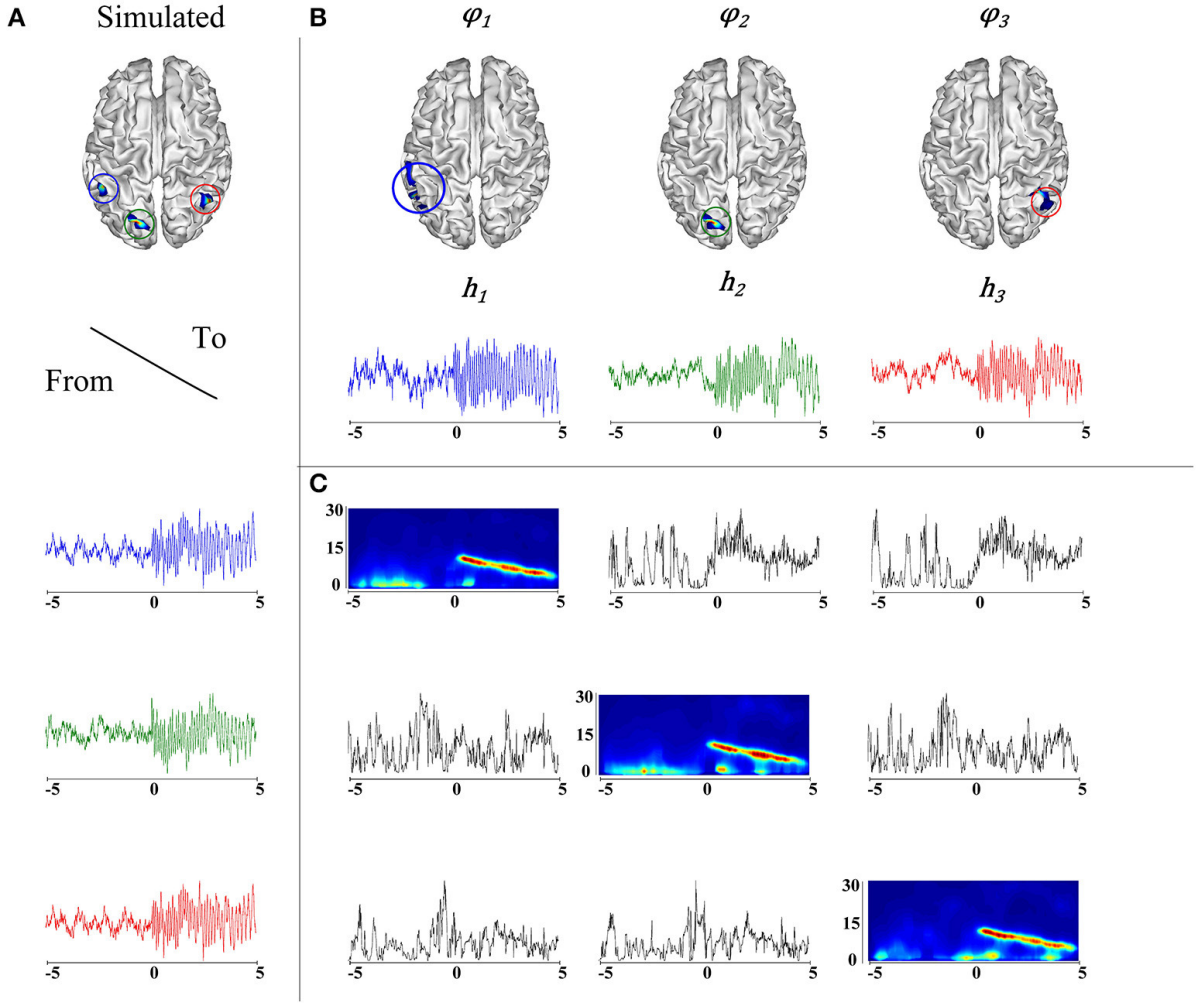
**(A)** Simulation of an epileptic network, in the top, locations of the simulated epileptic sources. In the bottom, their respective time courses. The seizure onset was simulated at *t* = 0 (blue). A fast propagation is simulated first to the green source and then to the red source. **(B)** Estimated ROIs and their respective time courses. **(C)** On the diagonal, the time-varying autospectra of the signals are depicted. The off diagonal plots express the information flow going from the signal indicated on the left to the signal indicated on the top.

It is worth noting that the correlation between the actual source locations (Figure 4A) and their corresponding *taMSP-FL-Conn* estimations (Figure 4B) is robust regardless of the considerable value of testing noise. Figure 4 explains this finding, showing an example of connectivity pattern (Figure 4C) accomplished by the 128-channels system after one random trial and zero-level SNR. As expected, the measured connectivity explains an increasing information flow from ROI 1 (marked in blue line) to the remaining ROIs. Nonetheless, this effect holds after the epileptic onset starts (i.e., *t* = 0). Before this moment, the connectivity behaves randomly, apparently, due to the simulated noise. Besides, the spectrogram, computed for the estimated time-courses, shows the epileptic activity with decreasing frequency behavior through time that starts at 12 Hz.

### 3.2. Performance examples on real-world neural activity application

#### EEG dataset of refractory focal epilepsy

Seizure data were collected from three patients (noted as P #1, P #2, and P #3, respectively) with refractory focal epilepsy (namely, temporal lobe epilepsy), who underwent pre-surgical evaluation at Ghent University Hospital and were subsequently treated with resective surgery. The ethical committee of Ghent University Hospital approved the study, and all patients gave written informed consent. The patients had been included in this study, according to the following criteria: (i) The patient was seizure free (i.e., Engel class 1) after surgery with minimum follow-up of 1.5 years, (ii) The electrode positions were known, (iii) The seizures and interictal spikes had the same lateralization in the EEG recordings, i.e., located on the left or right hemisphere, and (iv) there was no epileptic activity observed in routine EEG registrations after resection. Table 3 brings an overview of the patient information as described in detail by Strobbe et al. (2016).

**Table 3:** **Summary of the used patient data**.

**Patient**	**P #1**	**P #2**	**P #3**
Gender	F	F	F
Age (surgery)	41	26	64
Epilepsy type	TLE	TLE	TLE
# Electrodes	27	26 (27)	27
Removed elec.	–	O1	–
Sampling freq	128 Hz	256 Hz	128 Hz
Visual inspection	Left frontotemporal spikes + ictal discharges over the left frontotemporal region	Right frontotemporal spikes + bilateral frontotemporal ictal discharges	Left frontotemporal spikes + ictal discharges over the left frontotemporal region
# Spikes (avg)	35	41	14
Phase rev.	F7	F8	F7
MRI	Left hippocampal sclerosis	Dysplastic lesion in right gyrus temporalis inferior	Lesion in amygdala gyrus parahippocampalis
Surgery	Left selective amygdalohippocampectomy	Right anterior 2/3 temporal lobectomy	Left selective amygdalohippocampectomy
Resection vol.	4.7 cm^3^	32.7 cm^3^	6.1 cm^3^
Follow up	3 years	4 years	4 years
Engel class	Class 1	Class 1	Class 1

*F, Female; TLE, Temporal Lobe Epilepsy; Phase rev., Phase reversal; Elec. pos., Electrode Positions; N/A, not available*.

During acquisition, besides of the 21 electrodes that were located according to the International 10-20 system, three auxiliary inferior temporal electrodes were placed on each side of the head: zygomatic at F9-F10; Preauricular, T9-T10; and mastoid, Tp9-Tp10 positions. The electrode locations have been extracted from a computed tomography (CT) image acquired from the patients using a set of attached scalp electrodes.

As the processing stage, the sample frequencies of the EEG recordings are adjusted to 128 Hz for patients P #1 and P #3, while for patient P2 is 256 Hz. The data are common-average referenced and band-pass filtered between 0.5 and 40 Hz by using a zero-phase Butterworth filter to remove the baseline drift and reduce the high-frequency noise resulting from movement artifacts. An extra notch filter at 50 Hz is applied to filter out the power line noise. Independent Component Analysis (ICA) is employed to remove possible eyeblink and heartbeat artifacts. At the end of this stage, three seizures of P #1, two seizures of P #2 and one seizure of P #3 are used for the study. For each one of the seizures, the epoch is fixed as the window centered at the seizure onset time that is marked by an experienced epileptologist. Depending on the sample rate, the window length lasts either 5 or 10 s, aiming to account for the same number of time samples.

#### Implemented SOZ localization

The proposed methodology for SOZ localization, based on source connectivity analysis, comprises the following procedures:

– *Head model construction*: Individual head models are constructed for each patient by using the Finite Difference Method (FDM), making use of their presurgical anatomical MR images as performed in Strobbe et al. (2014). From the MR images, the nested meshes representing the scalp, outer skull, and inner skull are extracted and additionally converted to volumes by using the SPM Matlab toolbox. Further, we segment the gray matter, white matter, and cerebrospinal fluid (CSF), using the Freesurfer segmentation technique as in Fischl (2012). Employing these segmentations together with their inner volumes that are built from the surface meshes, 5-layered head models are constructed by including the scalp, skull, gray and white matter, and CFS layers. The CSF conductivity is set to 1.79 *S/m* (Baumann et al., 1997), 0.33 *S/m* for gray matter, 0.14 *S/m* for white matter, 0.022 *S/m* and 0.33 *S/m* for the skull and scalp, respectively (Montes-Restrepo et al., 2014; Vorwerk et al., 2014). Lastly, the resulting volumetric head models are resampled to 1×1×1 *mm* voxel resolution to compute the source space.
– *Source space calculation*: For each of the head models, the source space is constructed based on the segmented gray matter. The dipoles are assumed inside the gray matter (excluding the cerebellum) on a cubic grid equidistant to each other with a 1 mm spacing. Thus, we ensure that at least two voxels of gray matter are between the central node of the dipole model and the boundaries with other tissues in the *x*, *y*, and *z* directions. This setup results in about 10, 000 dipoles inside the gray matter for each model. We subsequently subsample the dipole source space with a spacing of 3 mm, yielding approximately 2, 000 dipoles inside the gray matter for each model as described in Strobbe et al. (2016).
– *Source reconstruction*: To estimate the cortical sources, we use the time adaptive version of the Multiple Sparse Volumetric Priors algorithm (Strobbe et al., 2014)^2^. The main variation of the volumetric MSP version is the incorporation of patches based on the neighboring information extracted from the gray matter, allowing the application of FDM forward models. Thus, the dipole orientations are determined according to the curvature of the segmented white matter to produce the volumetric priors (Phillips et al., 2002). Based on the dipole source space, we further construct a set of 256 volumetric regions of each patient, assuring a global gray matter coverage by randomly choosing a single dipole seed from all possible locations of 256 fixed gray matter volumes, covering the full gray matter. For each of the dipole seeds, a region is grown inside the gray matter of the patient so that the maximum distance to the original dipole and the smoothing factor are set to 5 and 0.6 mm, respectively. Thus, each of the regions is subsequently introduced into a single predefined covariance matrix, so that 256 covariance matrices are included as priors for inversion.
– *ROI and SOZ estimation*: We implement the *taMSP*, using 10 short time windows (lasting one second each one) that are in accordance with the same rule used for the simulated data. Then, a set of volumetric ROIs and their respective time series are estimated by all of the tested source connectivity methodologies. Lastly, the ROI with the highest outdegree or the highest power is marked as the SOZ target, depending on the tested source connectivity scenario.

#### Results of performed SOZ localization

**Figure 5:**
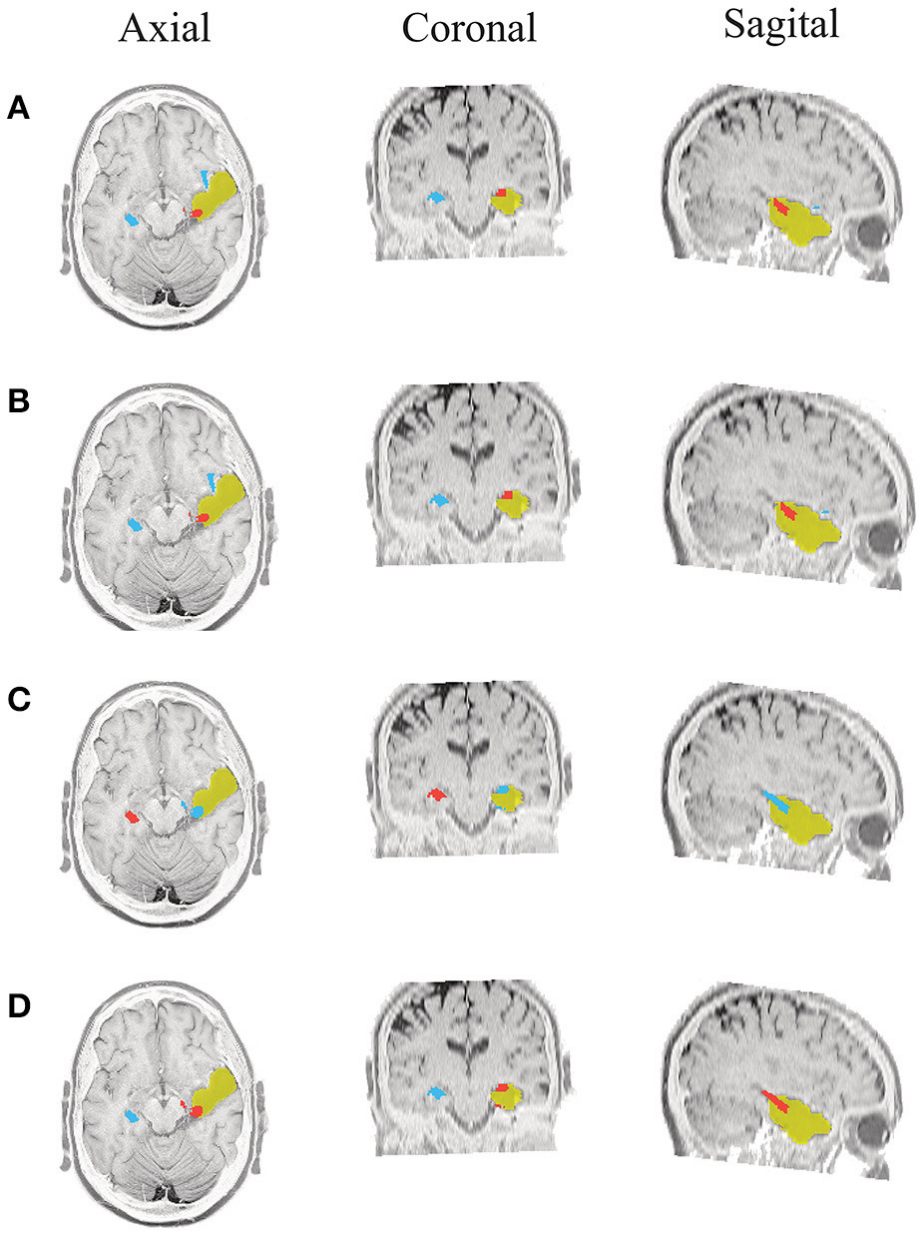
**Patient 1 seizure 1 (P1-1)**. Location of the resected zone (yellow patch), the estimated SOZ (red patch) and the remaining ROIs (blue patches) over the postsurgical MRI image. (A) taMSP-FL-Conn, **(B)** taMSP-Pow, **(C)** MSP-Av-Conn, **(D)** MSP-Pow.

**Figure 6:**
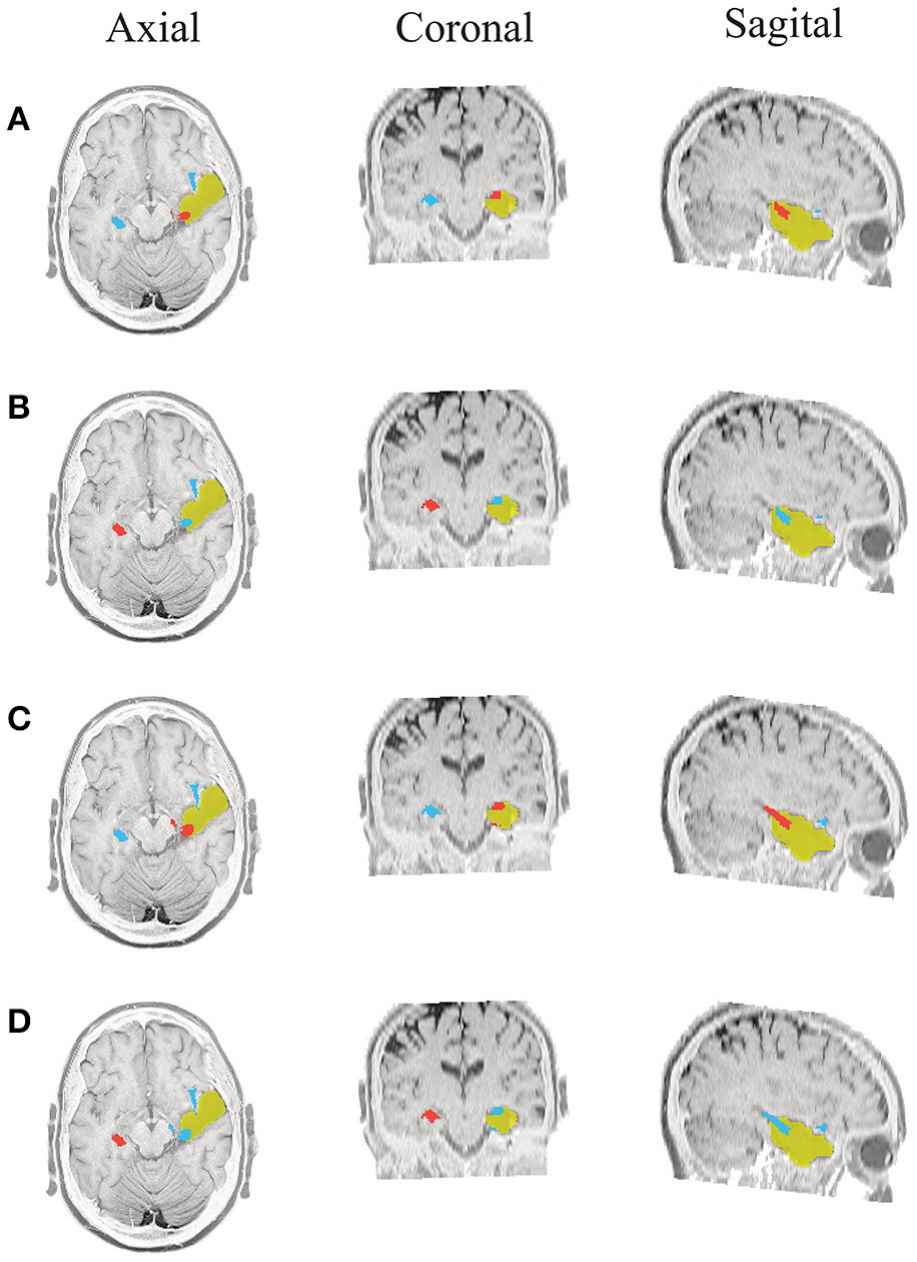
**Patient 1 seizure 2 (P1-2)**. Location of the resected zone (yellow patch), the estimated SOZ (red patch) and the remaining ROIs (blue patches) over the postsurgical MRI image. **(A)** taMSP-FL-Conn, **(B)** taMSP-Pow, **(C)** MSP-Av-Conn, **(D)** MSP-Pow.

**Figure 7:**
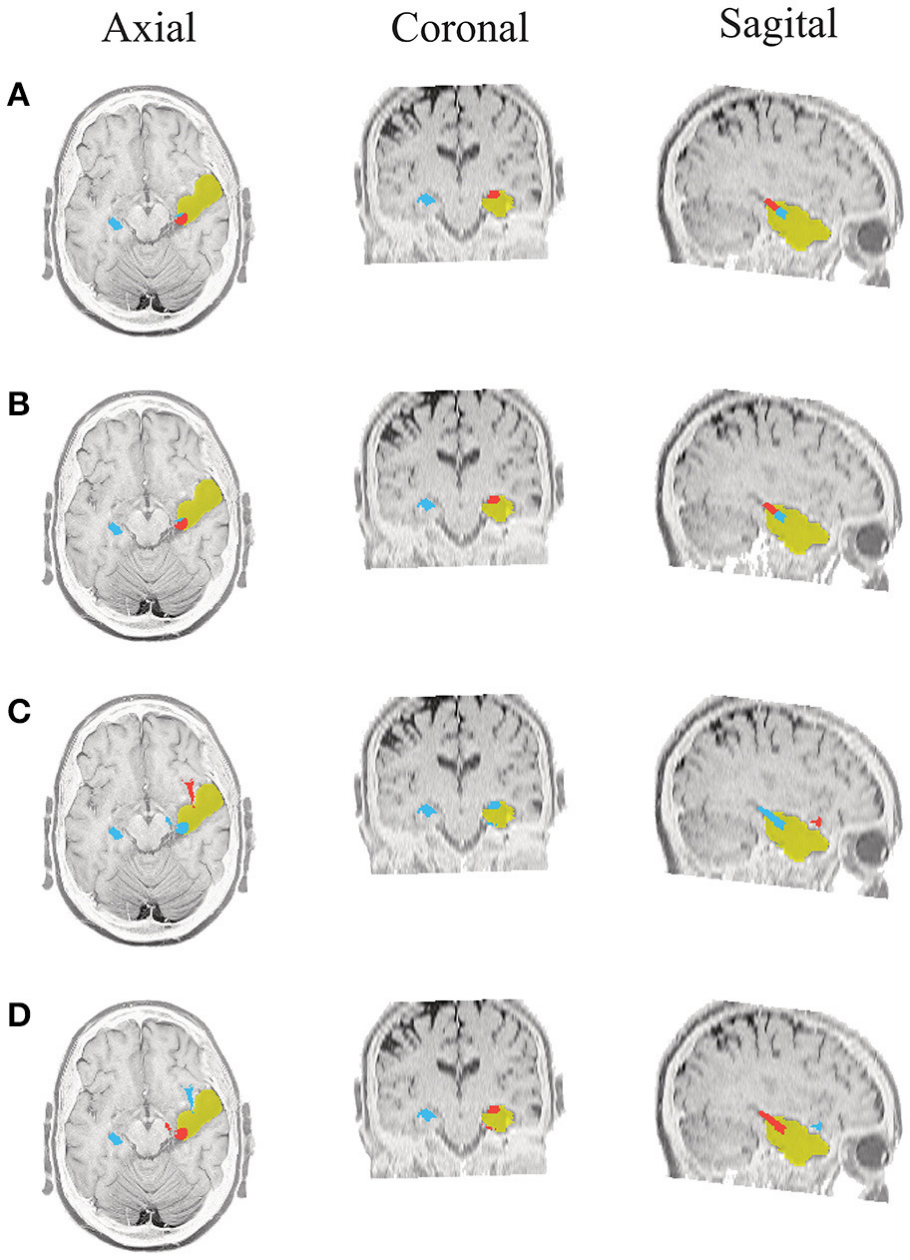
**Patient 1 seizure 1 (P1-3)**. Location of the resected zone (yellow patch), the estimated SOZ (red patch) and the remaining ROIs (blue patches) over the postsurgical MRI image. (A) taMSP-FL-Conn, **(B)** taMSP-Pow, **(C)** MSP-Av-Conn, **(D)** MSP-Pow.

**Figure 8:**
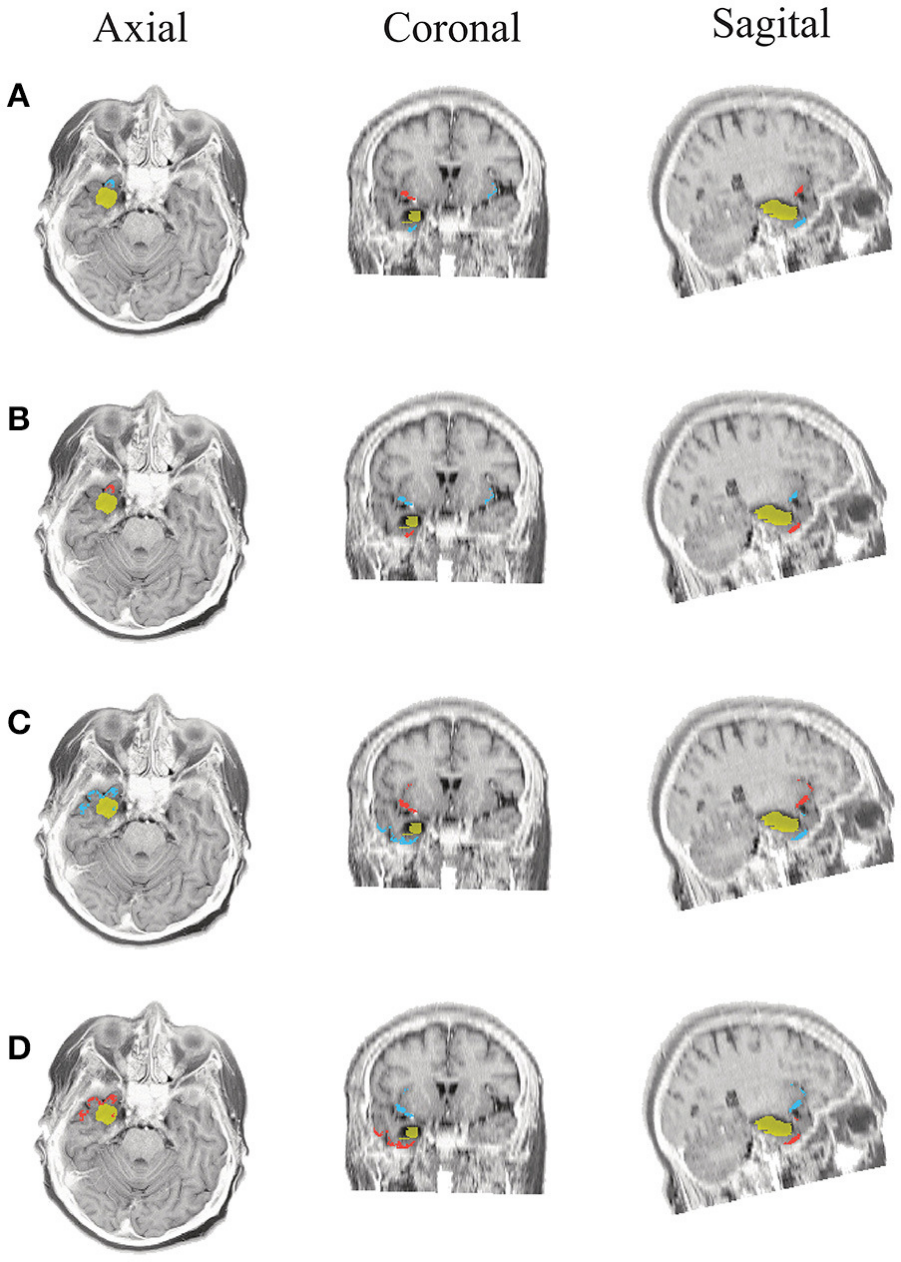
**Patient 2 seizure 1 (P2-1)**. Location of the resected zone (yellow patch), the estimated SOZ (red patch) and the remaining ROIs (blue patches) over the postsurgical MRI image. **(A)** taMSP-FL-Conn, **(B)** taMSP-Pow, **(C)** MSP-Av-Conn, **(D)** MSP-Pow.

**Figure 9:**
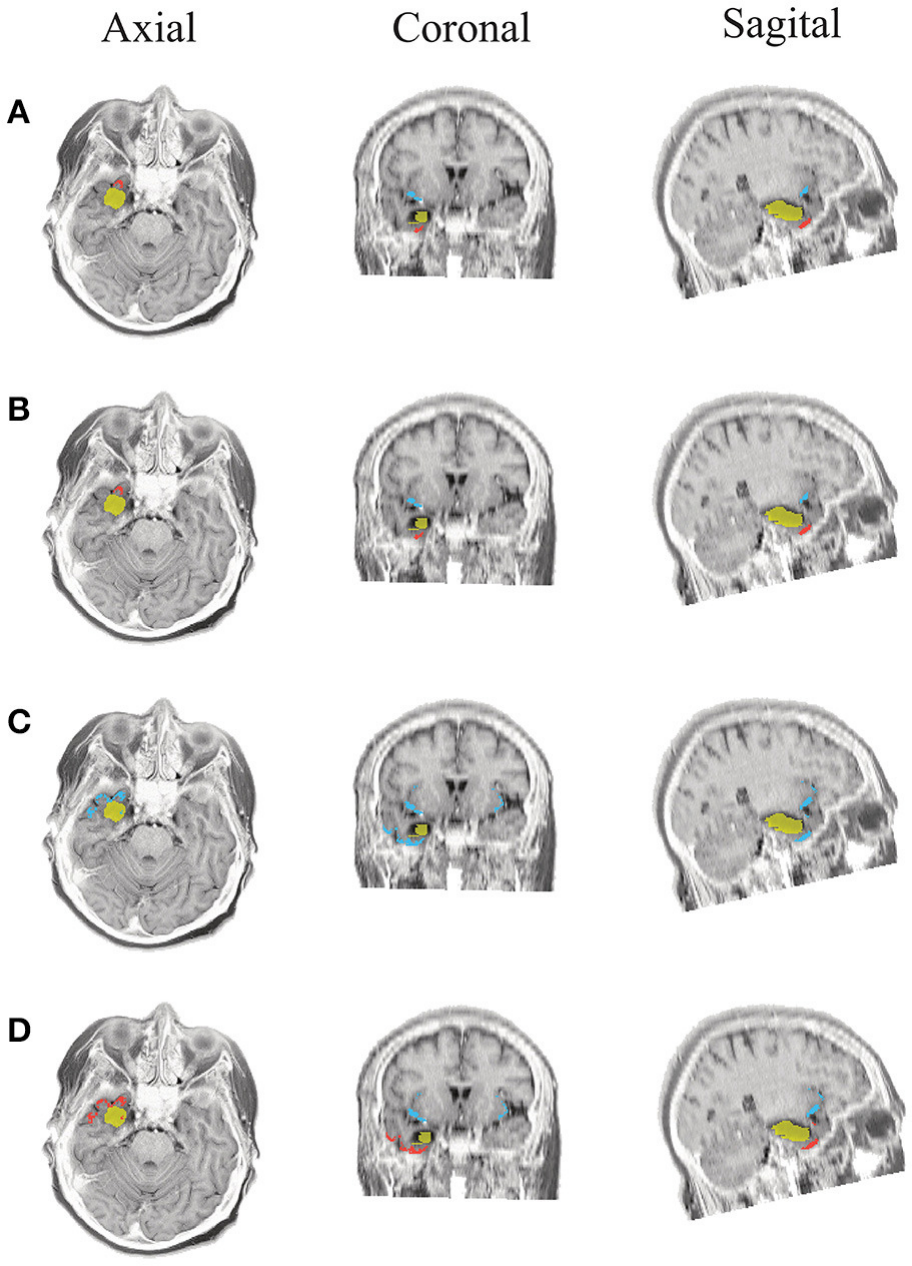
**Patient 2 seizure 2 (P2-2)**. Location of the resected zone (yellow patch), the estimated SOZ (red patch) and the remaining ROIs (blue patches) over the postsurgical MRI image. **(A)** taMSP-FL-Conn, **(B)** taMSP-Pow, **(C)** MSP-Av-Conn, **(D)** MSP-Pow.

**Figure 10:**
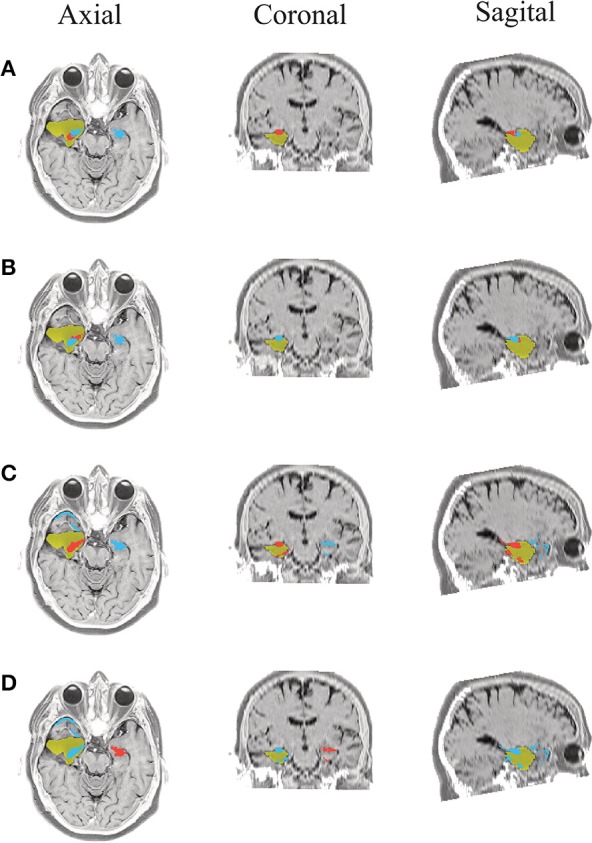
**Patient 3 seizure 1 (P3-1)**. Location of the resected zone (yellow patch), the estimated SOZ (red patch) and the remaining ROIs (blue patches) over the postsurgical MRI image. **(A)** taMSP-FL-Conn, **(B)** taMSP-Pow, **(C)** MSP-Av-Conn, **(D)** MSP-Pow.

Due to the postsurgical anatomical MR images are available for all admitted patients, we manually segment the resected zone to determine the volume of the resection. Consequently, for each tested methodology, we calculate the closest distance from the selected ROI (estimated SOZ) to the resection border. If the selected SOZ is detected in the resected zone, the distance is valued 0 mm. Figures 5–**10** illustrate the resected zone (yellow patch), estimated SOZ (red patch), and remaining nodes of the epilepsy network (blue patches) over three orthogonal slices of the postsurgical anatomical MR image. Note that these slices are visually chosen as the ones that better explicate the estimated brain activity, namely, the SOZ and the remaining ROIs.

As seen in Figures 5–7, all tested source connectivity scenarios estimate, at least, a single ROI within the resected zone in the case of Patient # 1. Consequently, their differences are observed just in the ROI time-course estimation and SOZ detection. Thus, the scenarios with SOZ detection based on power criteria (*taMSP-Pow* showed in panels B and *MSP-Pow* in panels D) fail in accurately localizing the SOZ target inside the resected zone in most of the recordings of this patient. Moreover, similar results are performed by *MSP-Av-Conn* that calculates the ROI time-courses by averaging the temporal patterns over the dipoles belonging to each ROI. Concerning *taMSP-FL-Conn*, it is the only scenario that localizes the SOZ target inside the resected zone for all the tested recordings of Patient # 1.

The results for Patient # 2 are similar to the ones obtained for Patient # 1 as seen in Figures 8, 9, though this case is the most challenging. Here, all the tested scenarios localize the ROI set, surrounding or with a few dipoles inside the resected zone. Moreover, Figure 10 shows that only *MSP-Pow* (the scenario without any temporal information) fails in detecting the SOZ target of Patient # 3, for which several ROIs are distinguished within the resected zone, improving the SOZ localization accuracy.

**Figure 11:**
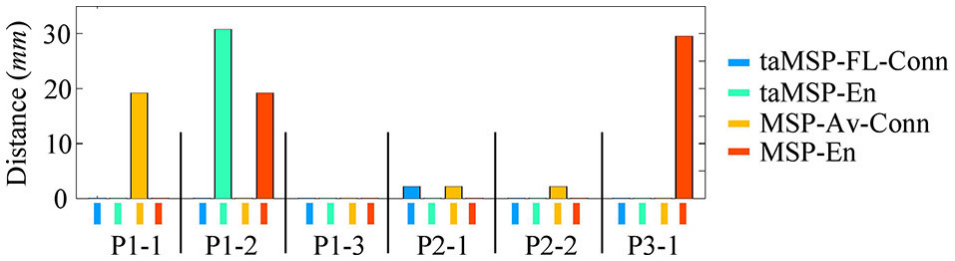
**Achieved distance to the resected zone for all the considered methods and seizures**.

On average, *taMSP-FL* estimates the lowest distance misalignment measured to the resected zone as shown in Figure 11 that summarizes the performance of each tested scenario of connectivity analysis for all patients. It is worth noting that there are not significant differences between the active areas achieved by either the *taMSP* and *MSP*, showing that the estimation of the ROI time-courses are crucial to properly conduct a source connectivity analysis. Summarizing, *taMSP-FL* provides the most confident distance computation since the SOZ target is always distinguished in a nearby region for several seizures of the same patient, unlike the remaining approaches that identify distant brain areas as the SOZ target in several recordings of each patient.

## 4. Discussion

With the aim to enhance the localization of seizure-onset zones, we propose a methodology that includes a time-varying source connectivity analysis, explicitly incorporating spatiotemporal information of the neural activity extracted from EEG recordings. In particular, we encode the source nonstationarities in basic processing stages: Inverse problem solution, estimation of ROI time courses, and connectivity assessment. In agreement with the above-obtained performance results, the following findings are worth mentioning.

### 4.1. Estimation of cortical sources

Several works have accepted that epilepsy-related brain activity poses a challenging problem due to its nonstationarity nature (Gonzalez Andino et al., 2001). In this regard, ESI solutions become especially hard when a large number of brain regions are simultaneously active, and they interact with each other, as it is the case for epilepsy networks. Generally, accurate localization of epilepsy network nodes strictly depends on the applied ESI solution. In this line of analysis, we have used the MSP algorithm that enables the reconstruction of superficial and deep sources (Friston et al., 2008). Thus, MSP embraces the entire cortical surface (both focal and deep) using a set of prior components, automatically identifying the patches that better reproduce the neural source distribution based on the available data covariance. Therefore, depending on the measured EEG data, MSP selects the model (sparse or distributed) to reconstruct the neural activity. Namely, if the source region is spread and superficial, the MSP solution fixes several prior components to emulate this kind of source activity. In contrast, if the source activity is deep and focal, just a few prior components are adjusted to describe the source distribution more accurately. As a consequence, the used ESI solution encourages a proper seedpoint for the ROI determination procedure in either case of source regions: sparse or distributed. However, several issues might reduce the performance of the involved ESI solution. In particular, an ESI algorithm is significantly harder to apply on ictal activity due to muscle and movement artifacts during seizures (Staljanssens et al., 2017), demanding a proper pre-processing stage of the examined EEG data. Also, the apparition of several brain regions, which are simultaneously active with different depths and extents, may degrade the localization accuracy. To this purpose, a fusion of several neuroimaging techniques as electro/magnetoencephalographic recordings would increase the performance of the ESI solution (Aydin et al., 2015).

With the aim to encode the strong spatiotemporal neural dynamics related to epileptic seizures, we carry out ESI through the proposed piecewise stationary source reconstruction. Hence, we use the time adaptive MSP solution that lies on two assumptions: EEG recordings are stationary within a short processing window, and a few neural sources produce the brain activity, yielding an enhanced (smooth, but localized) spatial resolution. As a result, the examples of the proposed method behavior performed on simulated and real-seizure EEG data shows that the *taMSP* strategy of training provides better accuracy and precision of SOZ localization than in the concrete case of comparison with the stationary *MSP* solution. This finding may be explained by the fact that the source analysis provided within a suitable short window allows decreasing the influence of noisy data, for instance, the one caused by EEG artifacts, inducing some ghost sources. Furthermore, from the performance results, we obtain that the achieved improvement in accuracy holds for *taMSP* within a wide SNR range, resulting in a robust source reconstruction tool. However, one aspect to ponder is the interval of the used short window for piecewise processing, which must be enough for dealing with a wide class of EEG artifacts that may appear.

Regarding the validation setup, we carry out the standard procedure used in numerous state-of-the-art works, employing the same source configuration to simulate and reconstruct the brain activity (see Table 1). However, this design (termed *inverse crime* Lucka et al., 2012) implies that the real current sources are also restricted to the locations of the chosen source space nodes, leading to overly optimistic results. To cope with this biasing issue, we have also designed a new simulation setup, having two different lead field matrices: one for simulation and another for reconstruction (see Section Appendix). From the obtained results for each setup, we show that our algorithm improves the SOZ localization, yielding differences between both scenarios according to the misalignment value in about 2 mm. This amount can be justified due to the discrepancy between the distances of the dipoles belonging to both meshes.

Another aspect to consider is the influence of the amount of EEG electrodes on the source reconstruction. As shown in Lantz et al. (2003) and Staljanssens et al. (2017), the number of electrodes affects the performance of source connectivity analysis. In simulations, we have shown that the proper insertion of temporal EEG nonstationarities might enhance the active source localization, resulting in an improved SOZ localization. However, to corroborate this finding, a most rigorous validation must be carried out, including simulations with more active sources (bigger than 3), and an increased number of real-world recordings in which we may properly examine how the EEG configuration affects the SOZ localization performance.

In respect of the used forward solution, for the sake of simplicity, we employ BEM models in the simulation setup, placing the sources on the brain cortex surface. Moreover, in real data, we employ more realistic FDM models, aiming to improve the ESI algorithm accuracy to identify epilepsy network nodes (ROIs). Nevertheless, we do not analyze the influence of the used forward model in the assessed connectivity. Consequently, we plan to address this issue in future works.

### 4.2. ROI identification

As a rule, the estimated source activity spreads over the entire cortical mesh so that the identification of spatially neighboring regions (that is, the ROI set) must be carefully performed. Furthermore, all ROIs must be densely allocated within small clusters to encode the spatial inhomogeneity of brain activity. Since epileptic events are not restricted to appear in very specific brain areas, we manage to detect the ROI set based on the estimated cortical sources. In this regard, we explore a strategy that relies on the neighboring information collected from the EEG forward problem to group the active dipoles more efficiently. As an advantage, this strategy performs compact-shape clusters, which are more similar to real scenarios. For instance, the estimated compact ROIs become desirable for dealing with epileptic seizures where there is not prior information about the sources to localize. Besides, the accomplished areas are small and homogeneous, promoting reliable connectivity calculation among networks.

### 4.3. Time-courses estimation of ROI sets

To the best of our knowledge, even though the quality of the ROI time courses highly affects the proper estimation of the connectivity among ROIs, there are few works devoted to properly carry out this task. Consequently, the most used procedure is to average the ROI time courses over the dipoles belonging to the specific ROI (Hassan et al., 2014). Nevertheless, the ROIs may contain dipoles with different dynamics, so that the average may yield a blurred or noisy time course representing the temporal behavior of such region. Another strategy is to compute pairwise connectivity between all the dipoles belonging to a pair of separated ROIs. Still, this strategy may increase the computational burden and may difficult the selection of the ROI that generates the seizure.

To cope with the above-mentioned issues, we proposed a new approach for estimating the particular dynamics of each ROI, in such a way, that a single time-courses can capture the time variant information of each particular area. To this end, we made the brain activity to appear solely inside the ROIs. Then, we estimated the ROI time courses by including a temporal penalty term that fosters the solution to remain almost constant within a close temporal neighborhood but varying along a large time horizon. This term allows the time courses estimation to track non-stationary activity present in EEG recordings. Obtained results for both simulated and real data have shown that the methodologies incorporating more precise estimation of the time-courses achieve better results than the methods that average time courses.

### 4.4. Time-varying assessment of directed functional connectivity

Several studies have addressed the problem of SOZ localization, using directed functional connectivity measures. The baseline approach can be the localization of ictal-onset zones based on Granger causality (Jung et al., 2011). However, its stationary premise obscures the detection of epileptic events. Among the available connectivity measures of information flow between neural networks, we implement a procedure, termed *swADTF*, that handles the non-stationary dynamics present on epileptic events (van Mierlo et al., 2011, 2013; Wilke et al., 2011). Consequently, *swADTF* tracks spectral changes over time at a particular frequency interval, through the TVAR model emphasizing those frequencies, which contribute the most to the signal power. Thus, we incorporated the connectivity measure to select the ROI in which the epileptic onset is more likely to be generated. Obtained results for both simulated and real EEG data show that methods using this connectivity measure perform better than the ones using a power-based criterion to select the onset epileptic location.

As regards the influence of the window length *T* on the outdegree computation. Here, we adjust the window length to near 10 seconds (centered at the seizure onset) that has been empirically fixed. As seen from Figure 4C, the connectivity behind the onset sample (estimated between the SOZ to the remaining ROIs set) tends to a constant value. In this regard, the window length *T* may be shortened down to the early time-points, but assuring that the onset is accurately fixed at the starting time point. In practice, there are some fluctuations in the labeled onset values, demanding to extend the window length for compensating the influence of the spurious estimates of connectivity computed ahead of the zero sample. As a future work, the analysis should be carried on a representative database, aiming to improve the window length estimation.

## 5. Conclusions

This work introduces a new method for source connectivity analysis that explicitly extracts temporal, spectral, and spatial information from EEG data to improve the SOZ localization. To this end, we initially carry out the time-varying brain source reconstruction, from which ROIs are localized as the brain areas with strong activity. As a means to compute further the corresponding ROI time courses, we also propose a new approach that relies on accurately tracking the non-stationary temporal dynamics in spatially focal brain areas. Aiming to encode the temporal dynamics of the underlying neural networks accurately, a directed functional connectivity measure is employed to quantify the information flow variations over the time window of interest. Obtained results on simulated and real EEG confirm that the proposed approach can localize the SOZ accurately.

As a future work, the authors plan to apply the method to more patients, including a more heterogeneous epilepsy population. Also, we will use the method for analyzing EEG recordings acquired under several stimuli to create brain-computer interfaces or analyzing patient emotional states.

## Author contributions

Conceived and designed the experiments: JM, PV, GS. Performed the experiments: JM, GS. Analyzed the data: JM, GS, PV, KV, GC. Contributed data/materials/analysis tools: JM, GS, PV, KV. Wrote the paper: JM, GC.

### Conflict of interest statement

The authors declare that the research was conducted in the absence of any commercial or financial relationships that could be construed as a potential conflict of interest.

## Figures and Tables

**Figure A1:**
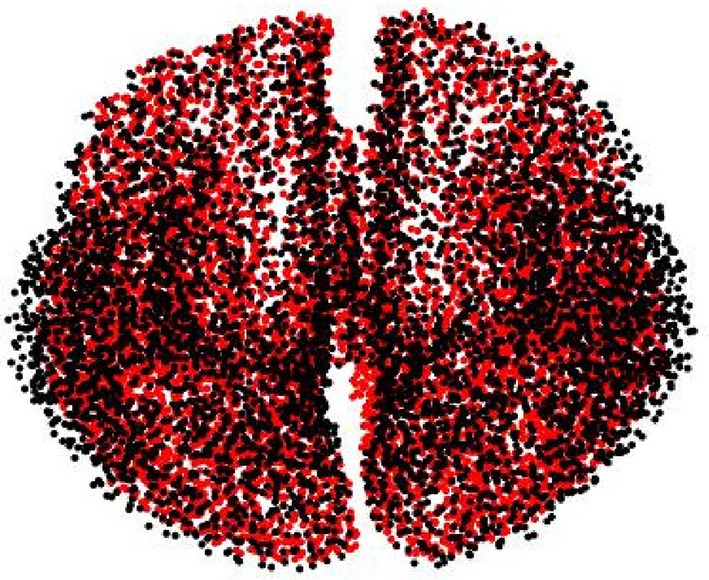
**Used meshes for simulating (black dots) and reconstructing (red dots) brain activity to avoid the inverse crime**.

**Figure A2:**
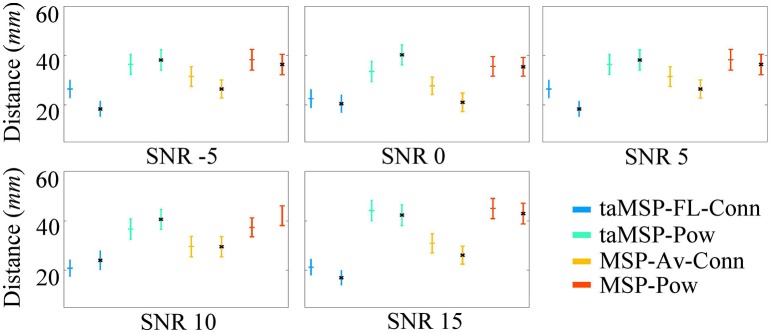
**Precision of the tested methodologies of SOZ location under several levels of noise, and avoiding the inverse crime**. The corresponding color marker for each scenario (i.e., blue for taMSP-FL-Conn) indicates the model with 32 channels, and the black marker (^*^) indicates the model with 128 channels.

## Footnotes

^1^http://www.fil.ion.ucl.ac.uk/spm.

^2^We denote the time adaptive Multiple Sparse Volumetric Priors as *taMSP* to avoid additional notations.

## Appendix

### Avoiding the “*inverse crime*”

This biasing effect is likely to occur when the lead field matrix used to generate the synthetic data is the same for estimating the reconstructed brain activity, leading to overly optimistic results due to the model and reality are identified (Kaipio and Somersalo, 2006). To evade this situation, we carry out the same experiments explained in Section 3.1, but modifying the lead field matrix used to simulate the EEG recordings, so that the real current sources are placed independently of the locations of the chosen source space nodes. To this, the volume conductor model for simulating the brain activity is constructed from a tessellated surface of the gray-white matter interface with 5, 124 vertices (possible source localizations), having source orientation fixed orthogonally to the surface. The gray-white matter interface is segmented from a different MRI, and the lead fields are calculated using the Boundary Element Method. Moreover, both meshes (simulation and reconstruction) are normalized to the MNI space. As a result, in average, the distance from a vertex of the simulation mesh to the closest vertex of the solution mesh is about 1.5 mm (see Figure A1).

Figure A2 displays the mean and standard deviation for the obtained misalignment, estimated after 100 trials at each SNR value in either EEG system configuration. As expected, when modifying the mesh to simulate EEG recordings, the results behave similarly to the validation layout shown in Table 1. Once again, our method (taMSP-FL-Conn) achieves the lowest misalignment rates for all the tested scenarios. Nevertheless, these rates increase in about 2 mm compared with the previous results. This difference can be explained because of the distance between the vertices of both meshes.

## References

[B1] Astolfi L. Cincotti F. Mattia D. De Vico Fallani F. Tocci A. Colosimo A.. (2008). Tracking the time-varying cortical connectivity patterns by adaptive multivariate estimators. IEEE Trans. Biomed. Eng. 55, 902–913. 10.1109/TBME.2007.905419 18334381

[B2] Aydin Ü. Vorwerk J. Dümpelmann M. Küpper P. Kugel H. Heers M.. (2015). Combined EEG/MEG can outperform single modality EEG or MEG source reconstruction in presurgical epilepsy diagnosis. PLoS ONE 10:e0118753. 10.1371/journal.pone.0118753 25761059 PMC4356563

[B3] Bastos A. M. Schoffelen J.-M. (2015). A tutorial review of functional connectivity analysis methods and their interpretational pitfalls. Front. Syst. Neurosci. 9:175. 10.3389/fnsys.2015.00175 26778976 PMC4705224

[B4] Baumann S. B. Wozny D. R. Kelly S. K. Meno F. M. (1997). The electrical conductivity of human cerebrospinal fluid at body temperature. IEEE Trans. Biomed. Eng. 44, 220–223. 10.1109/10.554770 9216137

[B5] Brodbeck V. Spinelli L. Lascano A. M. Wissmeier M. Vargas M.-I. Vulliemoz S.. (2011). Electroencephalographic source imaging: a prospective study of 152 operated epileptic patients. Brain 134, 2887–2897. 10.1093/brain/awr243 21975586 PMC3187544

[B6] Brookes M. J. O'Neill G. C. Hall E. L. Woolrich M. W. Baker A. Corner S. P.. (2014). Measuring temporal, spectral and spatial changes in electrophysiological brain network connectivity. Neuroimage 91, 282–299. 10.1016/j.neuroimage.2013.12.066 24418505

[B7] Chen X. Lin Q. Kim S. Carbonell J. G. Xing E. P. (2012). Smoothing proximal gradient method for general structured sparse regression. Ann. Appl. Stat. 6, 719–752. 10.1214/11-AOAS514

[B8] Elshoff L. Muthuraman M. Anwar A. R. Deuschl G. Stephani U. Raethjen J.. (2013). Dynamic imaging of coherent sources reveals different network connectivity underlying the generation and perpetuation of epileptic seizures. PLoS ONE 8:e78422. 10.1371/journal.pone.0078422 24194931 PMC3806832

[B9] Fischl B. (2012). Freesurfer. Neuroimage 62, 774–781. 10.1016/j.neuroimage.2012.01.021 22248573 PMC3685476

[B10] Friston K. Frith C. Frackowiak R. (1993). Time-dependent changes in effective connectivity measured with pet. Hum. Brain Mapp. 1, 69–79. 10.1002/hbm.460010108

[B11] Friston K. Harrison L. Daunizeau J. Kiebel S. Phillips C. Trujillo-Barreto N.. (2008). Multiple sparse priors for the M/EEG inverse problem. Neuroimage 39, 1104–1120. 10.1016/j.neuroimage.2007.09.048 17997111

[B12] Giraldo-Suarez E. Martinez-Vargas J. Castellanos-Dominguez G. (2016). Reconstruction of neural activity from EEG data using dynamic spatiotemporal constraints. Int. J. Neural Syst. 26:1650026. 10.1142/S012906571650026X 27354190

[B13] Gonzalez Andino S. Grave de Peralta Menendez R. Lantz C. Blank O. Michel C. Landis T. (2001). Non-stationary distributed source approximation: an alternative to improve localization procedures. Hum. Brain Mapp. 14, 81–95. 10.1002/hbm.1043 11500992 PMC6871930

[B14] Grosse-wentrup M. (2009). Understanding brain connectivity patterns during motor imagery for brain-computer interfacing, in Advances in Neural Information Processing Systems 21, eds Koller D. Schuurmans D. Bengio Y. Bottou L.(Vancouver, BC: Curran Associates, Inc.), 561–568.

[B15] Hassan M. Dufor O. Merlet I. Berrou C. Wendling F. (2014). Eeg source connectivity analysis: from dense array recordings to brain networks. PLoS ONE 9:e105041. 10.1371/journal.pone.0105041 25115932 PMC4130623

[B16] Haufe S. Ewald A. (2016). A simulation framework for benchmarking eeg-based brain connectivity estimation methodologies. Brain Topogr. 10.1007/s10548-016-0498-y. [Epub ahead of print]. 27255482

[B17] Heers M. Chowdhury R. A. Hedrich T. Dubeau F. Hall J. A. Lina J.-M.. (2016). Localization accuracy of distributed inverse solutions for electric and magnetic source imaging of interictal epileptic discharges in patients with focal epilepsy. Brain Topogr. 29, 162–181. 10.1007/s10548-014-0423-1 25609211

[B18] Henson R. Mattout J. Singh K. Barnes G. Hillebrand A. Friston K. (2007). Population-level inferences for distributed MEG source localization under multiple constraints: application to face-evoked fields. Neuroimage 38, 422–438. 10.1016/j.neuroimage.2007.07.026 17888687

[B19] Jung Y.-J. Kang H.-C. Choi K.-O. Lee J. S. Kim D.-S. Cho J.-H.. (2011). Localization of ictal onset zones in lennox-gastaut syndrome using directional connectivity analysis of intracranial electroencephalography. Seizure 20, 449–457. 10.1016/j.seizure.2011.02.004 21515079

[B20] Kaipio J. Somersalo E. (2006). Statistical and Computational Inverse Problems, Vol. 160 Berlin: Springer Science & Business Media.

[B21] Lantz G. De Peralta R. G. Spinelli L. Seeck M. Michel C. (2003). Epileptic source localization with high density EEG: how many electrodes are needed? Clin. Neurophysiol. 114, 63–69. 10.1016/S1388-2457(02)00337-1 12495765

[B22] Lucka F. Pursiainen S. Burger M. Wolters C. H. (2012). Hierarchical bayesian inference for the EEG inverse problem using realistic FE head models: depth localization and source separation for focal primary currents. Neuroimage 61, 1364–1382. 10.1016/j.neuroimage.2012.04.017 22537599

[B23] Montes-Restrepo V. van Mierlo P. Strobbe G. Staelens S. Vandenberghe S. Hallez H. (2014). Influence of skull modeling approaches on EEG source localization. Brain Topogr. 27, 95–111. 10.1007/s10548-013-0313-y 24002699

[B24] Pellegrino G. Hedrich T. Chowdhury R. Hall J. A. Lina J.-M. Dubeau F.. (2016). Source localization of the seizure onset zone from ictal EEG/MEG data. Hum. Brain Mapp. 37, 2528–2546. 10.1002/hbm.23191 27059157 PMC6867380

[B25] Phillips C. Rugg M. D. Friston K. J. (2002). Anatomically informed basis functions for EEG source localization: combining functional and anatomical constraints. Neuroimage 16, 678–695. 10.1006/nimg.2002.1143 12169252

[B26] Pittau F. Mégevand P. Sheybani L. Abela E. Grouiller F. Spinelli L.. (2014). Mapping epileptic activity: sources or networks for the clinicians? Front. Neurol. 5:218. 10.3389/fneur.2014.00218 25414692 PMC4220689

[B27] Schoffelen J.-M. Gross J. (2009). Source connectivity analysis with MEG and EEG. Hum. Brain Mapp. 30, 1857–1865. 10.1002/hbm.20745 19235884 PMC6870611

[B28] Staljanssens W. Strobbe G. Van Holen R. Birot G. Gschwind M. Seeck M.. (2017). Seizure onset zone localization from ictal high-density EEG in refractory focal epilepsy. Brain Topogr. 30, 257–271. 10.1007/s10548-016-0537-8 27853892

[B29] Strobbe G. Carrette E. Lpez J. D. Restrepo V. M. Roost D. V. Meurs A.. (2016). Electrical source imaging of interictal spikes using multiple sparse volumetric priors for presurgical epileptogenic focus localization. Neuroimage 11, 252–263. 10.1016/j.nicl.2016.01.017 26958464 PMC4773507

[B30] Strobbe G. van Mierlo P. Vos M. D. Mijovi B. Hallez H. Huffel S. V.. (2014). Multiple sparse volumetric priors for distributed EEG source reconstruction. Neuroimage 100, 715–724. 10.1016/j.neuroimage.2014.06.076 25014435

[B31] Tibshirani R. Saunders M. Rosset S. Zhu J. Knight K. (2005). Sparsity and smoothness via the fused lasso. J. R. Stat. Soc. Ser. B (Stat. Methodol.). 67, 91–108. 10.1111/j.1467-9868.2005.00490.x

[B32] van Mierlo P. Carrette E. Hallez H. Raedt R. Meurs A. Vandenberghe S.. (2013). Ictal-onset localization through connectivity analysis of intracranial EEG signals in patients with refractory epilepsy. Epilepsia 54, 1409–1418. 10.1111/epi.12206 23647147

[B33] van Mierlo P. Carrette E. Hallez H. Vonck K. Roost D. V. Boon P. Staelens S. (2011). Accurate epileptogenic focus localization through time-variant functional connectivity analysis of intracranial electroencephalographic signals. Neuroimage 56, 1122–1133. 10.1016/j.neuroimage.2011.02.009 21316472

[B34] Vorwerk J. Cho J.-H. Rampp S. Hamer H. Knösche T. R. Wolters C. H. (2014). A guideline for head volume conductor modeling in EEG and MEG. Neuroimage 100, 590–607. 10.1016/j.neuroimage.2014.06.040 24971512

[B35] Wilke C. Worrell G. He B. (2011). Graph analysis of epileptogenic networks in human partial epilepsy. Epilepsia 52, 84–93. 10.1111/j.1528-1167.2010.02785.x 21126244 PMC3200119

[B36] Wipf D. P. Owen J. P. Attias H. T. Sekihara K. Nagarajan S. S. (2010). Robust Bayesian estimation of the location, orientation, and time course of multiple correlated neural sources using MEG. Neuroimage 49, 641–655. 10.1016/j.neuroimage.2009.06.083 19596072 PMC4083006

